# Applicability of MDR1 Overexpressing Abcb1KO-MDCKII Cell Lines for Investigating In Vitro Species Differences and Brain Penetration Prediction

**DOI:** 10.3390/pharmaceutics16060736

**Published:** 2024-05-29

**Authors:** Emőke Sóskuti, Nóra Szilvásy, Csilla Temesszentandrási-Ambrus, Zoltán Urbán, Olivér Csíkvári, Zoltán Szabó, Gábor Kecskeméti, Éva Pusztai, Zsuzsanna Gáborik

**Affiliations:** 1 Charles River Laboratories Hungary, H-1117 Budapest, Hungary; emoke.soskuti@crl.com (E.S.); nora.szilvasy@crl.com (N.S.); csilla.temesszentandrasi-ambrus@crl.com (C.T.-A.); zoltan.urban@crl.com (Z.U.); oliver.csikvari@crl.com (O.C.); 2 Doctoral School of Semmelweis University, Molecular Medicine Division, H-1085 Budapest, Hungary; 3 Department of Medical Chemistry, Albert Szent-Györgyi Medical School, University of Szeged, H-6720 Szeged, Hungary; szabo.zoltan@med.u-szeged.hu (Z.S.); kecskemeti.gabor@med.u-szeged.hu (G.K.); 4 Department of Chemical and Environmental Process Engineering, Faculty of Chemical Technology and Biotechnology, Budapest University of Technology and Economics, H-1111 Budapest, Hungary; pusztai.eva@vbk.bme.hu

**Keywords:** BBB penetration, K_p,uu,brain_ prediction, MDR1, BCRP, ABCB1 knock-out, preclinical MDR1, species differences, MDR1 inhibitors

## Abstract

Implementing the 3R initiative to reduce animal experiments in brain penetration prediction for CNS-targeting drugs requires more predictive in vitro and in silico models. However, animal studies are still indispensable to obtaining brain concentration and determining the prediction performance of in vitro models. To reveal species differences and provide reliable data for IVIVE, in vitro models are required. Systems overexpressing MDR1 and BCRP are widely used to predict BBB penetration, highlighting the impact of the in vitro system on predictive performance. In this study, endogenous Abcb1 knock-out MDCKII cells overexpressing MDR1 of human, mouse, rat or cynomolgus monkey origin were used. Good correlations between ERs of 83 drugs determined in each cell line suggest limited species specificities. All cell lines differentiated CNS-penetrating compounds based on ERs with high efficiency and sensitivity. The correlation between in vivo and predicted K_p,uu,brain_ was the highest using total ER of human MDR1 and BCRP and optimized scaling factors. MDR1 interactors were tested on all MDR1 orthologs using digoxin and quinidine as substrates. We found several examples of inhibition dependent on either substrate or transporter abundance. In summary, this assay system has the potential for early-stage brain penetration screening. IC_50_ comparison between orthologs is complex; correlation with transporter abundance data is not necessarily proportional and requires the understanding of modes of transporter inhibition.

## 1. Introduction

Determining the brain exposure of drugs, especially of those with a central nervous system (CNS) target, is crucial for de-risking efficacy and toxicity issues early in drug discovery [1]. The brain is separated from the systemic circulation by the blood–brain barrier (BBB), a complex and dynamic interface with multiple roles. It is composed of brain microvascular endothelial cells connected by tight junctions, and supported by microglial cells, astrocytes, pericytes and the capillary basement membrane, adding up to a cellular membrane with constant thickness, limited pinocytotic activity and negative surface charge [2]. The transport of compounds across the BBB is tightly regulated, resulting in distinct pharmacokinetic properties of certain drugs in the brain compared to those in the blood. It involves ATP-binding cassette (ABC) transporter, multidrug resistance 1 (ABCB1/P-gp or MDR1), breast cancer resistance protein (ABCG2/BCRP) and multidrug resistance-associated protein 4 (ABCC4/MRP4). The major quantifiable solute carrier (SLC) transporters in brain microvessels are OAT3 (SLC22A8), GLUT1 (SLC2A1), LAT1 (SLC7A5) and MCT1 (SLC16A1) [3,4,5,6,7,8,9]. Of these, MDR1 is the most studied and most relevant gatekeeper [10,11].

Tools are available to determine the BBB penetration of drugs in development ranging from preclinical animal models to various in vitro and in silico tools, but every model has its limitations and translation to human is still challenging. Preclinical animals, mainly rodents, are used in neurotoxicity studies and to determine drug concentration in the brain [1]. The advantage of animal models is that they comprise all factors that influence the transport across the BBB, but because of substantial differences in the abundance and nature of transporters, translation to humans requires further investigation [1,12]. Despite the highly conserved nature of MDR1 [13], controversial data have been reported previously in vivo [14,15,16], underpinning the necessity for a set of in vitro assays enabling direct comparisons of MDR1 interactions across species. Hence, we aimed to create an in vitro test system to collect data about MDR1 from human and preclinical species on quantitative transport, affinity to various transporters and transporter abundance [1,4,5,6,7,12,17,18,19].

The major pharmacological factor that needs to be optimized in CNS drug discovery is K_p,uu,brain_ (unbound brain-to-plasma partition coefficient), since it is the unbound brain concentration that drives target binding and subsequent pharmacological response, according to the free drug hypothesis [10,20,21,22,23,24]. Higher K_p,uu,brain_ values of CNS drugs are preferable during drug development, as this will result in lower systemic toxicity concerns [25]. Considering recent efforts of the pharmaceutical industry to implement the 3R initiative to reduce preclinical animal experiments with more predictive in vitro tools, the improvement of in vitro in vivo extrapolation (IVIVE) is of high importance. Because of the complexity of the BBB, primary cell-based in vitro models are cost- and work-intense, and therefore less ideal for screening purposes. Systems overexpressing the two most important efflux transporters, MDR1 and BCRP, in all combinations have been evaluated previously by numerous groups. We learnt from these studies about the importance of the assay systems; the parental cell line and the transporter expression level have a significant influence on the predictive performance [17,26,27,28,29,30,31]. Our aim was to generate an in vitro system utilizing endogenous canine Abcb1-knock-out (KO) MDCKII cells overexpressing the MDR1 transporter of either human, mouse, rat or cynomolgus monkey origin to enable direct correlation of human and preclinical in vitro data. Bidirectional permeability and efflux ratio (ER) in the presence or absence of zosuquidar were determined for 83 drugs. In vivo rodent K_p,uu,brain_ data from the literature were compared to investigate whether the in vitro ERs determined in screening setup can be used for CNS permeability classification and quantitative K_p,uu,brain_ prediction. Our MCDKII-BCRP cell line, which has previously been shown to be useful in BBB penetration prediction [32,33], has been added to the screen, and combined MDR1 and BCRP substrate data were used for K_p,uu,brain_ prediction [17,28,29,30,31,34]. To compare data across species, transporter abundance was determined to calculate relative expression factors (REF), which are important input data for PBPK models as well. Inhibition was assessed in all cell lines with 21 compounds using two representative MDR1 substrates, digoxin and quinidine, to identify potential species differences in transporter specificity and sensitivity.

## 2. Materials and Methods

### 2.1. Materials

Reagents and non-radiolabeled chemicals were purchased from Merck/Sigma-Aldrich (St. Louis, MO, USA), Cayman Europe OÜ (Tallinn, Estonia), Selleck Chemicals (Houston, TX, USA), Biosynth (Compton, UK), Thermo Fisher Scientific (Waltham, MA, USA), Toronto Research Chemicals (Toronto, ON, Canada), Biogal Rt. (Debrecen, Hungary), VWR (Debrecen, Hungary) and MedChemExpress (Monmouth Junction, NJ, USA). All chemicals were of analytical grade. Ultima Gold XR scintillation fluid was obtained from PerkinElmer (Waltham, MA, USA). ^3^H-digoxin ([^3^H(G)], 23.8 Ci/mmol) and ^3^H-quinidine ([9-^3^H], 20 Ci/mmol) were from American Radiolabeled Chemicals (St. Louis, MO, USA). Stable isotope (^13^C and ^15^N) labeled proteospecific peptide fragments common for all orthologs of MDR1 (IATEAIENFR), ATP1A1 (IVEIPFNSTNK) and dog specific MDR1 (FYDPLAGSVLIDGK) were ordered from JPT Peptide Technologies (Berlin, Germany) in the form of tagged SpikeTides™ TQL peptides.

### 2.2. Cell Line Generation and Culture Conditions

Cell lines were generated as described previously in [PMID: 36901890]. In brief, sequence-verified cDNA encoding human MDR1 (NCBI Reference Sequence: NM_000927.4), rat Mdr1a (NM_133401.1), mouse Mdr1a (NM_011076.3) and cynomolgus monkey Mdr1 (NM_001287322.1) was synthesized by GenScript. Transduced and antibiotic-selected Abcb1KO-MDCKII cells were subjected to single cell cloning by calcein-AM-based FACS, and amplified clones were functionally tested for transporter-specific efflux activity. The best-performing clones were selected for continued validation, and are hereafter referred to as the hMDR1, mMDR1, rMDR1, and cyMDR1 cell lines. Empty vector-transduced Abcb1KO-MDCKII-Mock cells were used as control. Wild type parental MDCKII cells were transduced with sequence-verified cDNA (GenScript) encoding human BCRP (NCBI Reference Sequence: NM_004827.1). The maintenance and seeding of cells are described in detail [35]. Briefly, Abcb1KO-MDCKII-MDR1 and Mock, and MDCKII-BCRP, cells were cultured in DMEM with high glucose, supplemented with 10% fetal bovine serum, 2 mM GlutaMAX^TM^, 100 units/mL penicillin and 100 µg/mL streptomycin at 37 °C, 5% CO_2_, and 90% relative humidity. For transport experiments, cells were seeded on Millicell^TM^ high-pore-density 0.4 μm PCF 96 well cell culture plate inserts (Millipore, Merck KGaA, Darmstadt, Germany) at a density of 25,000 cells/well and grown for 5 days at 37 °C in an atmosphere of 5% CO_2_ and 95% relative humidity. The culture medium was changed once, the day before the experiment.

### 2.3. Bidirectional Transport Assays

Transport assays were performed as previously described [36] with minor modifications. After washing and preincubation for 15 min with prewarmed Hanks’ balanced salt solution (HBSS) at pH 7.4, the experiment was started at t = 0 by replacing HBSS in the donor compartment (either apical or basolateral) with HBSS containing the substrate (1 µM) or the mixture of substrate and inhibitors (zosuquidar, 1 or 5 µM for MDR1 or Ko143, 1 µM for BCRP). The final concentration of DMSO was ≤0.1% in all transport buffers. Samples were taken from both the receiver and donor sides at 120 min to determine the recovery in addition to the transport activity. Samples and dosing solutions were diluted twofold in methanol and analyzed by LC-MS/MS. In inhibitory studies, A-B and B-A permeabilities of ^3^H-digoxin (1 µM, 0.17 µCi/mL) and ^3^H-quinidine (0.1 or 1 µM, 0.17 µCi/mL) probe substrates were applied in the absence or presence of increasing concentrations of the selected inhibitors at predetermined timepoints by cell lines (digoxin—120 min; quinidine—30 min (rMDR1); hMDR1, mMDR1 and cyMDR1—60 min). To determine the amounts of radiolabeled substrates (digoxin or quinidine) transported, samples mixed with Ultima Gold XR liquid scintillation cocktail were measured with a MicroBeta2 microplate counter (PerkinElmer). The tightness of the cell monolayer was controlled via the permeability of Lucifer Yellow (LY, 40 µg/mL). Experiments showing LY permeation higher than P_app_ 2 × 10^−6^ cm/s were rejected.

### 2.4. Analytical Measurements

Sample analysis was performed on an LS-I autosampler (Sound Analytics, Niantic, CT, USA) equipped with Agilent 1260 HPCL pumps coupled to a Sciex 6500+ Triple Quadrupole Mass Spectrometer (AB SCIEX, Framingham, MA, USA). Chromatographic separation was achieved on a Phenomenex Kinetex F5 column (30 × 2.1 mm, 2.6 µm, Phenomenex Inc., Torrance, CA, USA) with gradient elution starting from 2% B eluent with a flow rate of 0.7 mL/min, holding for 0.12 min, followed by a linear gradient from 2 to 95% B in 0.48 min, then holding at 95% B for 0.1 min with a flow rate of 1 mL/min, then a re-equilibration of the column with 2% B with 1 mL/min for 0.3 min. Eluent A consisted of 0.1% formic acid in water, eluent B consisted of 0.1% formic acid in acetonitrile. Then, 10 µL samples were injected into the HPLC-MS/MS system. Mass spectrometric detection was performed in SRM mode, and ion transitions for each analyte were optimized during method development. Samples were analyzed in pools, with each pool containing three to four analytes.

### 2.5. Quantitative Targeted Absolute Proteomics (QTAP)

Absolute protein expression levels (pmol/mg membrane protein) of target transporters were determined via a PRM analysis in the NanoLC–MS/MS using the peptide probes to quantify the target molecules. MDR1 was quantified from filter-grown hMDR1, mMDR1, rMDR1a and cyMDR1 cell lines, and BCRP from MDCKII-BCRP cell monolayers. Cell culturing and seeding conditions were equivalent to those in bidirectional transport assays. The membrane protein fractions of the cells were enriched using ProteoExtract^TM^ Native Membrane Protein Extraction kit (Merck) according to the manufacturer’s protocol. The membrane-enriched buffer II fractions were used for further analysis. The protein content of the samples was determined using BCA Protein Assay kit (Thermo Fisher Scientific) following the manufacturer’s acetone precipitation protocol. For all the samples, 10 µg protein was processed with an On Pellet Digestion protocol. The samples were reduced with 20 mmol DTT at 60 °C for 30 min and alkylated with 40 mmol IAA in the dark at room temperature for 30 min. The protein content was precipitated by adding seven volumes of ice-cold acetone and incubated at −20 °C overnight. After centrifugation at 15,000× *g* for 10 min, at 4 °C, the supernatant was discarded. The protein pellet was washed three times with 0.5 mL acetone/water (85/15, *v*/*v*) mixture. After centrifugation at 14,000× *g* for 10 min at 4 °C, the protein pellet was dissolved in 15 µL RapiGest SF Surfactant (Waters, Milford, MA, USA) and was incubated at 100 °C for 5 min. After cooling to room temperature, 55 µL 100 mmol AmBic (pH = 8), 10 µL IS peptide mix (1 pmol/peptide) and 0.25 µg/5 µL trypsin were added to the mixtures. The samples were incubated at 37 °C for 30 min and another 0.25 µg/5 µL trypsin was added, and the mixture was digested at 37 °C for 5.5 h. Digestion was stopped by the addition of 1 µL concentrated FA. The resulting peptide samples were purified using Pierce™ C18 Spin Tips (Thermo Fisher Scientific) according to the manufacturer’s instructions, with an additional detergent removal step. This was achieved by washing the tips with 2 × 50 µL DCE after the prescribed desalting step. The samples were then evaporated under vacuum, then resolved in 90 µL of the initial eluent. The samples were centrifuged with 10,000× *g* for 10 min at 4 °C and 5 µL of the supernatant was injected into NanoLC–MS/MS. NanoLC-MS/MS analysis was carried out on a Waters ACQUITY UPLC M-Class LC system (Waters) coupled with an Orbitrap Exploris™ 240 mass spectrometer (Thermo Fisher Scientific). A Symmetry^®^ C18 (100 Å, 5 µm, 180 µm × 20 mm) trap column was used for trapping and desalting the samples. The chromatographic separation of peptides was accomplished on an ACQUITY UPLC^®^ M-Class Peptide BEH C18 analytical column (130 Å, 1.7 µm, 75 µm × 250 mm) at 45 °C by gradient elution. Water (solvent A) and acetonitrile (solvent B), both containing 0.1% FA, were used as mobile phases at a flow rate of 350 nL/min. The sample temperature was maintained at 5 °C. The mass spectrometer was operated using the equipped Nanospray Flex Ion Source. The parallel reaction monitoring (PRM) method was used to monitor the *m*/*z* transitions for the + 2 charged peptides precursors of interest. To reach the maximum sensitivity, precursor ions were fragmented using optimal collision energies. The automatic gain control (AGC) setting was defined as 1 × 10^5^ charges, the maximum injection time was set to auto, and resolution was set to 15,000. Data acquisition was performed using Xcalibur^TM^ 4.6 (Thermo Fisher Scientific), and Skyline 22.2.1.278 [37] was used for data evaluation. The ratio of the peptides to stable isotope labeled internal standard was used for protein quantification. Final quantitative results are shown as the ratio to total protein content injected into NanoLC-MS/MS (pmol protein/mg total membrane protein).

### 2.6. Data Analysis

(a) Bidirectional transport assays

All the experiments were performed in three biological replicates and repeated two or three times; each data point corresponds to the mean of at least six values.

Apparent permeability (P_app_), ER and mass balance (recovery) were determined as has been published [36]. Compounds with lower than 60% recovery were excluded from the data analysis. The half-maximal inhibitory concentration (IC_50_) is used as a measure of inhibitory drug potency. If inhibition did not exceed 50% at the highest inhibitor concentration tested IC_50_ calculations were not performed, and the highest applied concentration was used for correlation. IC_50_ values were derived from a four-parametric logistic equation (log(inhibitor) vs. response–variable slope); the curve was fitted to the ER vs. inhibitor concentration plot using non-linear regression in GraphPad Prism version 9.0 (GraphPad, La Jolla, CA, USA). In the comparison studies, using linear regression, the coefficient of determination (R^2^) was determined with GraphPad Prism version 9.0. The difference from the line of identity was quantified with residual standard error (RSE), which was calculated as follows:

(1) $$RSE=\sqrt{\frac{\sum_{i}^{n}{\left(x_{i}-y_{i}\right)}^{2}}{n}}$$

where x_i_ and y_i_ represent the calculated ER or the IC_50_ of the component i in substrate or inhibition studies, respectively, and n represents the number of data points.

(b) Predictive Performance Metrics

MDR1 and/or BCRP substrate properties were investigated by binary classification analysis on CNS+ and CNS− compounds. The possible outcomes are the following: True positive (TP), when the CNS− compound showed impaired brain distribution due to MDR1 and/or BCRP efflux mechanisms. True negative (TN), where there was no efflux and the CNS+ compound crossed the BBB. False negative (FN), when a compound showed impaired brain penetration, but not due to an efflux mechanism. False positive (FP) compounds are efflux transporter substrates that do not show impaired brain distribution. The sensitivity and specificity of the estimation of brain penetration along with MDR1 and BCRP activity were calculated from predictive performance metrics.

(c) Calculations of predicted unbound brain-to-plasma partition coefficient (K_p,uu,brain_)

First, NET ER was calculated and used for human predictions,

(2) $$NET\;ER={ER}_{\left(-inh\right)}-{ER}_{\left(+inh\right)}$$

where ER_(−inh)_ is ER in the absence of inhibitor, and ER_(+inh)_ is ER in the presence of inhibitor, for hMDR1 (ER_MDR1_) or hBCRP (ER_BCRP_).

Total ER comprising both hMDR1 and hBCRP activities is

(3) $$Total\;ER=\left({NET\;ER}_{MDR1}\right)+\left({NET\;ER}_{BCRP}\right)+1$$

REF was calculated as follows [38]:

(4) $$REF=\frac{transporter\;abundance\;in\;brain\;capillaries\left(pmol/mg\;protein\right)}{transporter\;abundance\;in\;transporter\;overexpressing\;cell\;line\left(pmol/mg\;protein\right)}$$

The data used for REF calculation are shown in Table S2.

Equation (3) modified with REF:

(5) $${Total\;ER}_{REF\;corrected}=\left({NET\;ER}_{MDR1}\right)\times {REF}_{MDR1}+\left({NET\;ER}_{BCRP}\right)\times {REF}_{BCRP}+1$$

Predicted K_p,uu_,_brain_ calculated with four different methods, listed here (Equations (6)–(9)).

1. Predicted K_p,uu_,_brain_ based on hMDR1 ER was calculated as follows:

(6) $$K_{p,uu,brain}=\frac{1}{NET\;ER\times REF+1}$$

2. Predicted K_p,uu,brain_ calculated from total ER is as follows:

(7) $$K_{p,uu,brain}=\frac{1}{Total\;ER}$$

3. Predicted K_p,uu,brain_ calculated from total ER corrected with relative expression levels of MDR1 and BCRP is as follows:

(8) $$K_{p,uu,brain}=\frac{1}{{REF}_{MDR1}\times {(NET\;{ER}_{MDR1}}_{.}{)+{REF}_{BCRP}\times (NET\;ER}_{BCRP})+1}$$

4. K_p,uu,brain_ calculated with α and β scaling factors, with a modification of Equation (8), is as follows:

(9) $$K_{p,uu,brain}=\frac{1}{\alpha \times {(NET\;{ER}_{MDR1}}_{.}{)+\beta \times (NET\;ER}_{BCRP})+1}$$

where α and β represent scaling factors determined with non-linear least squares regression using the Levenberg–Marquardt algorithm, detailed below.

(d) Estimation of α and β scaling factors for K_p,uu,brain_ prediction

Non-linear least squares regression using the Levenberg–Marquardt algorithm was applied to estimate the α and β parameters of the model. The regression was performed in RStudio (version 2023.12.0) using the nls_multstart() function from the nls.multstart package [39], and the best fit was determined based on the best Akaike information criterion (AIC). Residual plots were used to assess the goodness of fit for each model (Figures S1 and S2).

The dataset of 55 compounds (atenolol, mannitol, sumatriptan have been excluded) was used for the first fit. On the residual plots (Figure S1), four compounds had extremely high residuals compared to the others (standardized residuals were 2 or higher). Investigating these outliers, we observed that their K_p,uu,brain_ values were much higher than 1. Since the mathematical formula used for K_p,uu_,_brain_ calculation is incapable of predicting K_p,uu,brain_ values higher than 1.2, we excluded these compounds from the regression analysis. A new model was fitted omitting these data and the parameters were re-estimated accordingly. Since these new residual plots were appropriate (Figure S2), the re-estimated model parameters were accepted.

## 3. Results

### 3.1. Substrate Screen

#### 3.1.1. Comparing the Functional Activity of hMDR1, rMDR1, mMDR1 and cyMDR1 Cell Lines

**Figure 1:**
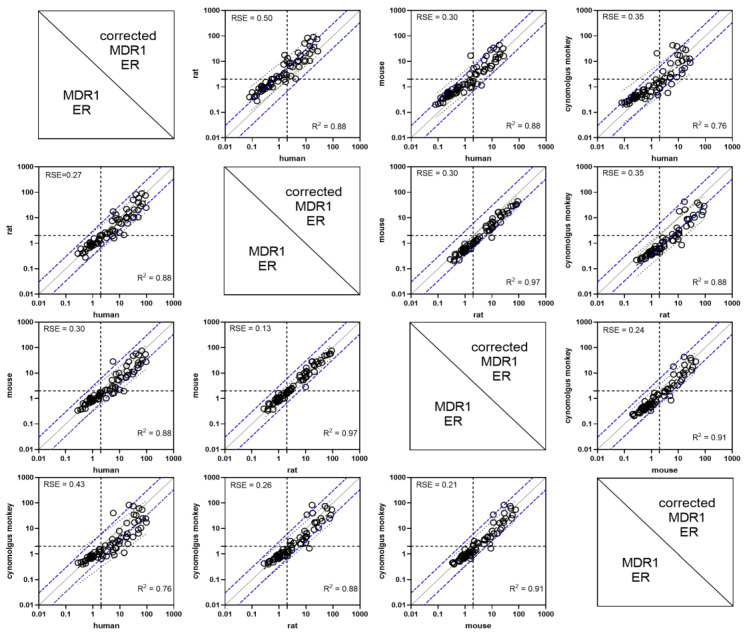
ERs across MDR1-expressing cell lines for a set of selected compounds were positively correlated. Graphs in the upper right triangle show transporter abundance-corrected ERs, while the lower-left triangle represents determined ERs. Solid lines show the line of identity. The densely dotted lines represent three-fold difference from the line of identity. ER values of 2 are used as the cut-off to classify MDR1 substrates (dotted horizontal and vertical lines).

First, MDR1-overexpressing cells were fully validated using two prototypical MDR1 substrates, digoxin and quinidine, and assay parameters for substrate and inhibitor assesment were defined accordingly. For substrate screen 83 proprietary compounds, including both CNS+ and CNS− drugs, were selected, and in vitro ERs were assessed across hMDR1, rMDR1, mMDR1 and cyMDR1 as well as mock control cell lines (1 µM, 120 min, ± zosuquidar). Using the same conditions across substrates allowed for the proper comparison of ERs determined (Figure 1, Table S1). Among the 83 compounds, 47 were known hMDR1 substrates. Using an ER cut-off value of 2, according to the regulatory guidelines (ICH M12), we identified 47, 43, 40 and 36 substrates of hMDR1, rMDR1, mMDR1 and cyMDR1, respectively. The ER in mock cells for all compounds were close to unity. Famotidine was applied as a permeability control; its passive permeability was comparable across cell lines.

MDR1 ERs calculated for this set of compounds across cell lines were positively correlated: rMDR1 vs. mMDR1 (R^2^ = 0.97, *p* < 0.001); cyMDR1 vs. mMDR1 (R^2^ = 0.91, *p* < 0.001); cyMDR1 vs. rMDR1 (R^2^ = 0.88, *p* < 0.001); hMDR1 vs. rMDR1 (R^2^ = 0.88, *p* < 0.001); hMDR1 vs. mMDR1 (R^2^ = 0.87, *p* < 0.001). Surprisingly, the weakest correlation was seen between hMDR1 and cyMDR1 (R^2^ = 0.76; *p* < 0.001) (Figure 1).

Next, to correct for transporter expression differences between the cell lines, ERs were corrected by transporter abundance (Table S2). Correlation analysis between the transporter abundance-corrected ERs was run between each pair of MDR1 orthologs. (Figure 1, upper right triangle). This correction changed the slope of the fitted line to deviate more from the line of identity. Of note, this analysis was run for all drugs including non-substrates and species-specific substrates. Remarkably, if we analyzed only shared substrates of orthologs, correction with transporter abundance improved the correlation (The data presented in this study are available on request from the corresponding author). To measure standard deviation from the line of identity, residual standard errors (RSE) were calculated.

Remarkably, the dispersion of ER values differs across the four orthologs, so we calculated interquartile ranges (IQR) for each dataset. Human MDR1 has, by far, the largest IQR (16.57), and hence, the widest dynamic range, suggesting the highest sensitivity for substrate recognition. Rat and mouse MDR1 data had similar interquartile ranges (8.92 and 8.09, respectively), and cyMDR1 (4.85) showed the smallest dispersion of ER values. The human MDR1 cell line showed the highest MDR1 activity for most tested compounds, which aligns well with the highest transporter expression level. This shows that transporter protein abundance is not proportionate in all cases with the ER; still, human MDR1 proved to be the best in revealing weak MDR1 substrates. However, the ER of cimetidine and trimethoprim was the highest in rat, paclitaxel and ritonavir ER were higher in rat and mouse MDR1, daunorubicin was higher in cyMDR1, mitoxantrone and saquinavir ER were the lowest in hMDR1, and the ER of vinblastine and ondansetron were comparable in all cells, suggesting potential species differences.

#### 3.1.2. Brain Permeability Classification Based on In Vitro ER

**Figure 2:**
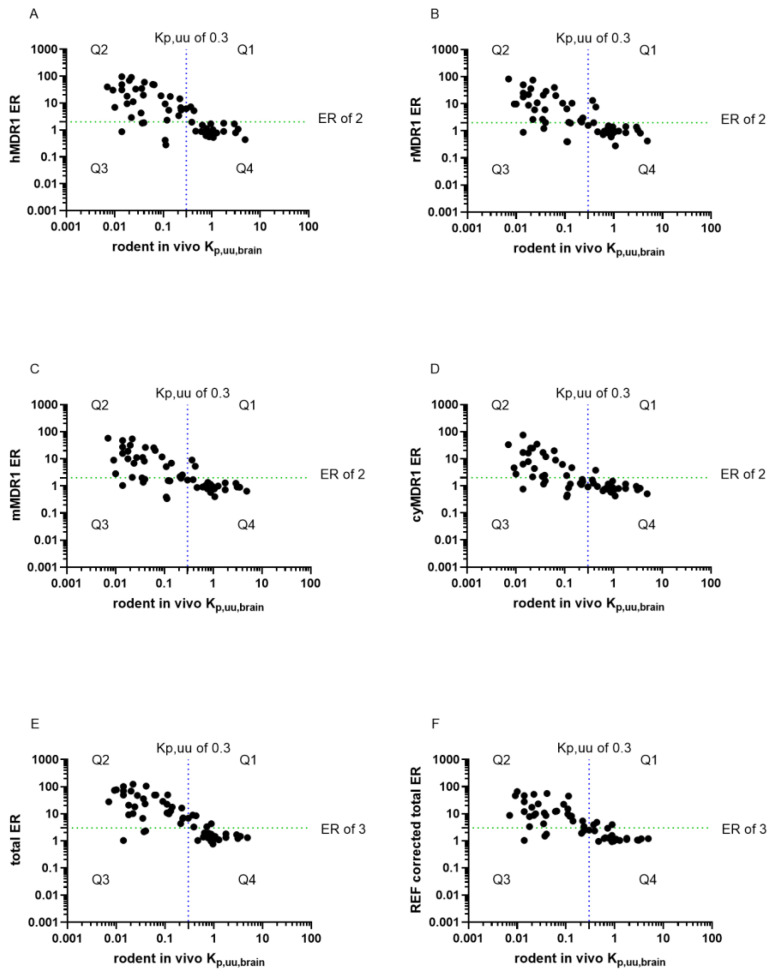
Correlation of rodent K_p,uu,brain_ with ER for 58 selected compounds. ER values were determined in hMDR1 (**A**), rMDR1 (**B**), mMDR1 (**C**) and cyMDR1 (**D**) cell lines or calculated as total ER (**E**) and REF-corrected total ER (**F**) of hMDR1 and hBCRP. Cut-offs of 2 and 3 were used for MDR1 ERs and for total ERs, respectively. A threshold of 0.3 was used for K_p,uu,brain_. The quadrants illustrate true negative (Q4), true positive (Q2), false positive (Q1) and false negative (Q3) predictions.

To identify the correlation between the in vivo rodent K_p,uu,brain_ and ERs, 58 compounds with known K_p,uu,brain_ were selected. The correlation between the K_p,uu,brain_ in rodents and in vitro ERs is shown in Figure 2. An ER of 2 was used as a cut-off to classify MDR1 substrates and a K_p,uu,brain_ of 0.3 was used as a threshold for CNS penetrant compounds. The resulting graphs are thus divided into four quadrants. Brain penetration (K_p,uu,brain_) was used as the logical value true condition, with compounds having K_p,uu,brain_ ≤ 0.3 considered positive. Q2 and Q3 comprise molecules with impaired brain penetration. Drugs in Q2 (true positives, TP) represent the subset of MDR1 substrates, where efflux activity likely limits brain penetration. Drugs in Q3 (false negatives, FN) have limited brain penetration but are unlikely to be MDR1 substrates. Drugs in Q1 (false positives, FP) are MDR1 substrates, but their brain penetration is not limited, probably due to other mechanisms, such as uptake or high passive permeability overwrite efflux, e.g., for trimethoprim and ondansetron [40,41]. Molecules in Q4 (true negatives, TN) can freely access the brain (Figure 2). Predictive performance metrics were calculated to show the efficacy of the established cell lines in BBB permeability classification (Table 1). Sensitivity refers to the ability of the model to correctly classify CNS-restricted molecules based on MDR1 substrate nature. We found that the human and rat MDR1 cell lines have the highest resolution to differentiate compounds with a sensitivity ≥80% (Figure 2A,B, respectively), while the sensitivity of the mouse and cynomolgus monkey MDR1 cell lines are lower; 71% and 65%, respectively (Figure 2C,D). Specificity was above 80% for all tested cell lines.

Subsequently, for a more reliable prediction of brain penetration, data generated in the hBCRP cell line were included in the analysis. hBCRP assay conditions and evaluations were identical to that of MDR1 cell lines, which allowed proper comparisons (Figure 2E,F). Total ER was calculated by combining ERs determined in BCRP and hMDR1 cell lines based on Equation (3) [42], with an ER cut-off of 3. The predictive performance of this total ER scored better than hMDR1 alone with respect to sensitivity, as out of the five false negatives, only three remained in this category—atenolol, mannitol and sumatriptan—revealing low passive permeability and subsequent limited brain penetration. Entacapone and indomethacin were first identified as false negatives using the hMDR1 screen alone, but as they are BCRP substrates, here they turned into true positives. However, the specificity of the total ER prediction is lower (79%), resulting in five false positives (ondansetron, trimethoprim, warfarin, etoricoxib, guanabenz). Eventually, total ERs were also assessed with the Relative Expression Factor (REF) (Equation (4); Figure 2E,F). REF is an in vitro and in vivo correlation scaler, calculated from transporter protein abundances in brain microvessels versus overexpressing cell lines [4,5]. Remarkably, metrics calculated without REF showed better prediction in all aspects than those with transporter abundance correction. Taken together, among all approaches, the data clearly show that total ER without REF correction had the highest sensitivity (91%) in identifying CNS compounds.

#### 3.1.3. Comparison of In Vitro Predicted K_p,uu,brain_ and In Vivo Rodent K_p,uu,brain_

The quantitative prediction of BBB penetration from physicochemical parameters is challenging even with the use of in silico models, and needs further refinement, e.g., by using in vitro efflux transporter data [10,43,44]. Numerous methods have been published on K_p,uu,brain_ prediction based on in vitro data, of which MDR1 ER is indispensable [10,17,28,29,30]. It needs to be emphasized that the goodness of predictions depends on in vitro assay properties [10,45,46]. Therefore, we were eager to see to what extent our cell lines would predict in vivo brain penetration data.

**Figure 3:**
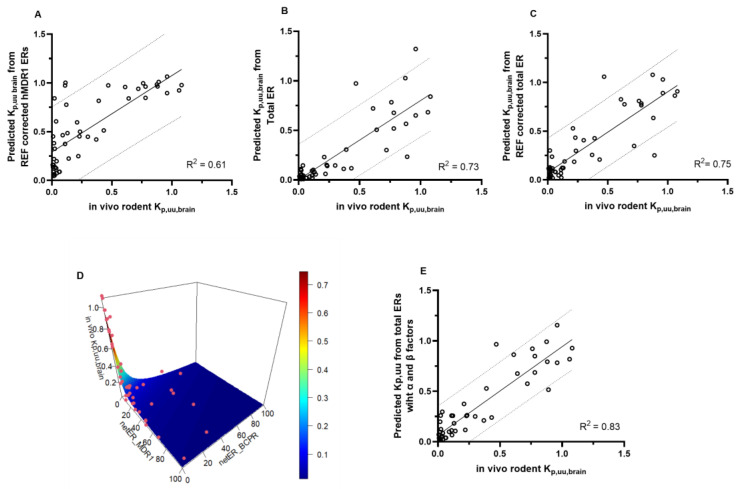
Prediction of K_p,uu,brain_ using ERs of hMDR1 and hBCRP cell lines. The solid lines represent best fit from simple linear regression analysis, while the dotted lines show the 95% prediction band of the best fit line. Atenolol, mannitol, sumatriptan and compounds with in vivo K_p,uu,brain_ > 1.2 were excluded from analysis. (**A**) Correlation between in vivo rodent K_p,uu,brain_ and predicted K_p,uu,brain_ from REF-corrected hMDR1 ERs. (**B**) Correlation between in vivo rodent K_p,uu,brain_ and predicted K_p,uu,brain_ from total ER. (**C**) Correlation between in vivo rodent K_p,uu,brain_ and predicted K_p,uu,brain_ from REF-corrected total ER. (**D**) Visual representation of data used for the estimation of α and β parameters. (**E**) Correlation between K_p,uu,brain_ calculated according to Equation (9) with the estimated α and β scaling factors and in vivo K_p,uu,brain_.

For this quantitative prediction, we calculated in vitro K_p,uu,brain_ with four different methods using ERs obtained in our hMDR1 and hBCRP cell lines. First, we calculated K_p,uu,brain_ from hMDR1 ERs according to Equation (5). Next, K_p,uu,brain_ values were calculated from either total ERs or REF-corrected total ERs according to Equations (6) and (7), to account for the difference between in vitro in vivo transporter abundance. Finally, to further improve the prediction performance of our data and achieve the best fit to in vivo K_p,uu,brain_, we used selected compounds to define α and β factors according to Equation (9) [28,29]. Three compounds (atenolol, mannitol and sumatriptan) with relatively low passive permeability and ER < 2 were excluded from the correlation, since one known limitation of these simple prediction models is that they do not account for the passive permeability of substances. BDDCS classification can be incorporated into the pipeline in a multi-step approach, as published previously [46]. The calculated in vitro K_p,uu,brain_ were compared against the corresponding in vivo rodent K_p,uu,brain_ data. The least promising correlation was seen using only the human MDR1 data (Figure 3A), where R^2^ was 0.61. The prediction was more accurate using both hMDR1 and hBCRP-derived total ER, as it improved the fitting to R^2^ = 0.73 (Figure 3B). The REF-corrected prediction was similar, with R^2^ = 0.75 (Figure 3C). The strongest correlation was observed between the predicted and in vivo K_p,uu,brain_ using the estimated α and β factors in the equation (Table 2), with R^2^ being 0.83 (Figure 3D,E).

#### 3.1.4. Species- and Substrate-Specific Differences in IC_50_ Values

First, kinetic parameters in hMDR1, rMDR1, mMDR1 and cyMDR1 cell lines were determined for both substrates, digoxin and quinidine, and accordingly the final concentrations of digoxin and quinidine were set as 1 µM, except for cyMDR1, for which a lower Km was estimated, necessitating a quinidine concentration of 0.1 µM. Inhibition potencies were assessed for 21 known MDR1 interactors (Table S3), including the two probe substrates, digoxin and quinidine, and species- and substrate-specific differences were analyzed. IC_50_ values were calculated from the ERs [47] determined at seven concentrations and normalized to solvent control. To investigate whether observed differences among species are due to different abundances of MDR1 proteins, IC_50_ values were normalized, and protein-corrected and non-corrected IC_50_ values were systematically compared. Of the 21 compounds, 18 inhibited the MDR1-mediated transport of probe substrates in a consistent and reproducible manner, and were analyzed; ritonavir and talinolol had no inhibitory effect on either substrate. Also, consistently with previous results, digoxin did not inhibit the transport of quinidine. In cases where the IC_50_ values were given as “greater than” due to solubility issues, the highest tested concentration was used as a surrogate for inhibition potential comparison. The IC_50_ values obtained cover three orders of magnitude demonstrating a wide dynamic range for the assays.

The species-specific MDR1 transport activity for digoxin and quinidine were assessed by ERs calculated and averaged from all the vehicle controls of the inhibition studies. Interestingly, the efflux activity of human MDR1 toward quinidine was significantly higher than for digoxin (47.3 ± 16.6 vs. 17.7 ± 5.9), while such substrate-specific differences were not seen in the other cell lines (cyMDR1, 10.7 ± 1.4 vs. 13.3 ± 4.4; rMDR1, 16.7 ± 4.8 vs. 17.2 ± 4.9; mMDR1, 15.9 ± 3.8 vs. 13.9 ± 3.6).

**Figure 4:**
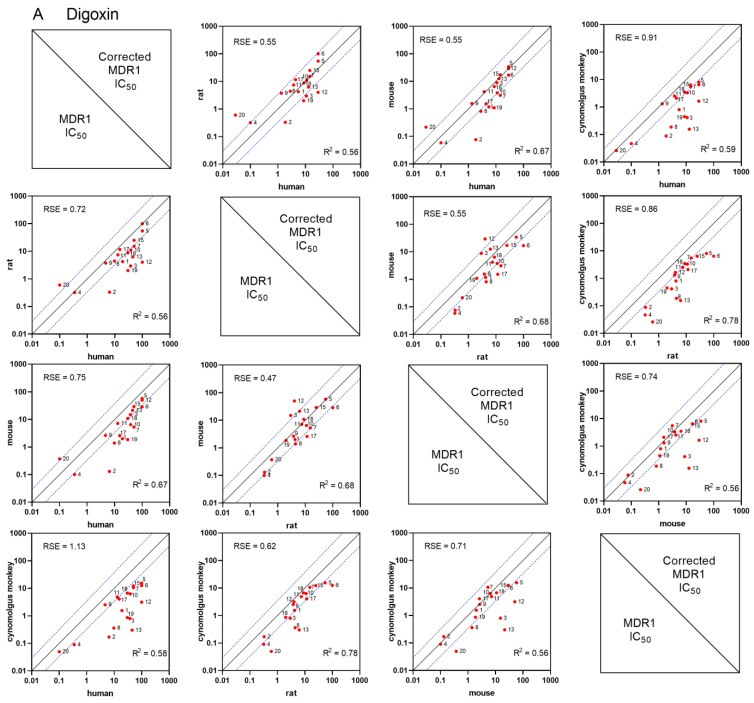

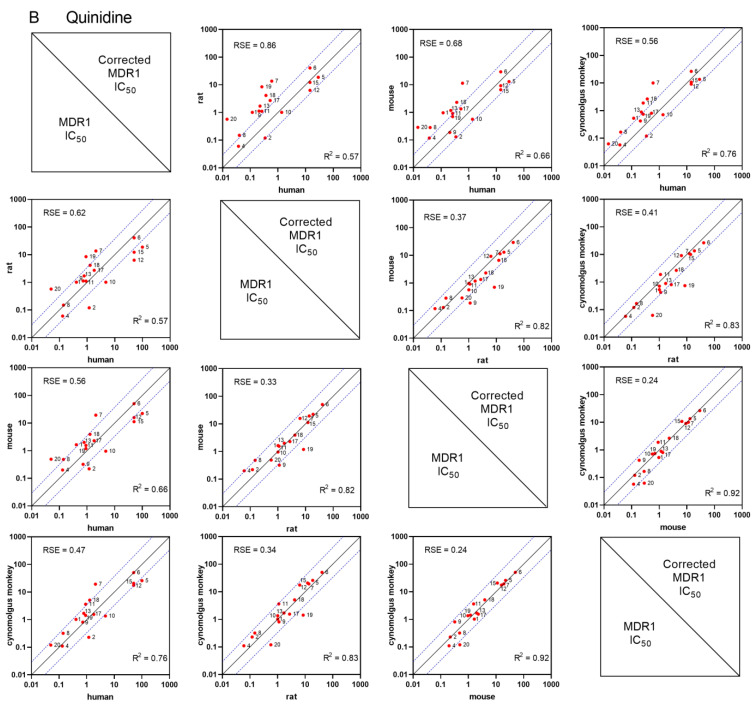
Correlation of IC_50_ values from human, rat, mouse or cynomolgus monkey MDR1 using digoxin (**A**) and quinidine (**B**) as probe substrates. R^2^ values have been determined for each pair using the logarithmically transformed IC_50_ values. Considering all correlations, all *p*-values of Pearson correlation were lower than 0.0079. RSE values were calculated against the line of identity and are shown on each graph. Red dots represent the means of at least two independent experiments, each with three technical parallels. Densely dotted lines show the 3-fold difference from the line of identity. Compounds are marked with numbers (Table S3). Graphs in the upper right triangle show transporter abundance-corrected IC_50_, while the lower left triangle represents uncorrected IC_50_ values.

**Figure 5:**
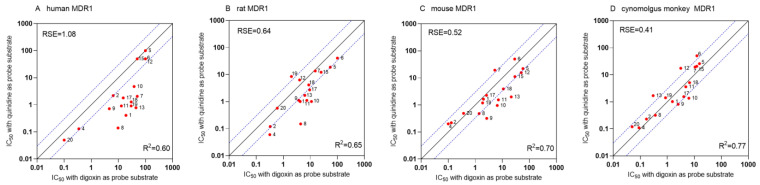
Correlation of IC_50_ values determined with either digoxin or quinidine as probe substrates in human (**A**), rat (**B**), mouse (**C**) or cynomolgus monkey (**D**) MDR1 cells. R^2^ values have been determined for each pair using the logarithmically transformed IC_50_ values. Considering all correlations, all p-values of Pearson correlation were lower than 0.0030. RSE values were calculated against the line of identity and are shown on each graph. Red dots represent the means of at least two independent experiments, each with three technical parallels. Densely dotted lines show the 3-fold difference from the line of identity. Compounds are marked with numbers (Table S3).

The correlation between IC_50_ values determined for each ortholog for digoxin and quinidine are shown in Figure 4A,B, respectively. Based on the linear regression, the strongest correlation (R^2^ = 0.78) was found between rat and cynomolgus MDR1 IC_50_ values for digoxin. To quantify differences, residual standard errors (RSE) were calculated against the line of identity, where lower RSE values correspond to smaller differences. The lowest RSE (0.47) was found between mouse and rat MDR1 with digoxin as the substrate. Interestingly, while IC_50_ values from human and cynomolgus MDR1 correlate well using quinidine, this correlation is weaker with digoxin. Overall, data indicate that the inhibition of digoxin resulted in higher IC_50_ values for human MDR1 than for the other investigated orthologs. No such pattern was observed using quinidine as a substrate. Correction with the transporter abundance has no effect on R^2^ but on the slope of the regression line. Protein correction resulted in lower RSE for all comparisons using digoxin, in contrast to other orthologs. Using quinidine as a probe, correction with transporter abundance resulted in higher RSE values in all cases. Comparing IC_50_ values between digoxin and quinidine for each MDR1 ortholog highlighted substrate-specific differences, especially for hMDR1 (Figure 5), where digoxin transport inhibition resulted in much higher IC_50_ values than that of quinidine. This tendency was also observed for mouse and rat MDR1, but to a lesser extent. The IC_50_ values determined in cyMDR1 cells correlated with an R^2^ of 0.77.

Next, we examined trends in IC_50_ values across compounds between orthologs. A ≥3-fold ratio between any two IC_50_ values was considered to be a real difference. The same set of IC_50_ comparisons were run after normalization for transporter expression. For elacridar, ketoconazole and zosuquidar IC_50_s are in the same range for all MDR1 orthologs using either digoxin or quinidine, independent of transporter abundance. Similarly, zosuquidar’s IC_50_ values are comparable, with the highest potency for human and cynomolgus MDR1 in both substrates.

For some compounds, such as the high-affinity MDR1 substrate verapamil, there were notable differences in IC_50_s for digoxin across the cell lines. The verapamil IC_50_ in the hMDR1 cell line was 15–35-fold higher than in rat, mouse and cyMDR1 cell lines. With quinidine, there was no difference in verapamil IC_50_ values, except for rMDR1, where the IC_50_ value was 6–9 times higher than for other orthologs. While protein-corrected IC_50_ values resulted in even more pronounced differences for quinidine, with digoxin, these differences diminished. Carvedilol, isradipine and tolvaptan inhibited digoxin and quinidine transport with the same pattern as verapamil. Digoxin transport inhibition by CSA yielded >20-fold (20–50-fold) higher IC_50_ values for hMDR1 than for other MDR1 orthologs. This difference was within 5–10-fold when using quinidine as the substrate. When comparing CSA IC_50_ values across cell lines, the differences between the two substrates were less than 3-fold, except for hMDR1, which showed a 5-fold difference. For high-affinity human MDR1 substrate loperamide, higher IC_50_ values were measured for both substrates in human MDR1. Correction with transporter abundance diminished these differences. IC_50_ values for human, rat and mouse MDR1-mediated digoxin or quinidine transport inhibition could not be calculated for etoricoxib (hMDR1), felodipine (hMDR1, mMDR1, rMDR1), saquinavir (hMDR1) or isradipine (hMDR1).

## 4. Discussion

This report is the first to investigate MDR1 activity in human as well as three preclinical species in the context of CNS penetration using transporter-overexpressing endogenous MDR1-knockout MDCKII cell lines, thus allowing side-by-side study of individual MDR1 orthologs. Despite recent advances in in silico and in vitro models, K_p,uu,brain_ prediction is still challenging, making preclinical studies unavoidable. Although in silico models using molecular descriptors and in vitro parameters exist, their predictive performance is moderate [44]. One challenge is to account for the role of transporters at the BBB [43]; further improvement of these models requires a better understanding of transporter–drug interactions and their impact on BBB penetration [10,44,48]. Inherent differences in cell lines lead to confounding results [10,45,46], likely because parental cells differ in tightness and transporter abundance; factors that make MDCK cells and higher transporter expression preferable [10,29,49,50,51,52,53]. Our cell lines fulfill these requirements, in that endogenous MDR1 activity is abolished, and relatively high MDR1 expression is achieved.

For the majority of the 83 compounds analyzed, comparable ERs were found across cell lines, with no notable outliers. The best correlation was observed between rMDR1 and mMDR1 (R^2^ = 0.96), in accordance with the published data [54]. Correlations between human vs. rat and human vs. mouse MDR1 (R^2^ = 0.88 for both) are comparable to previous results [49]. The weakest correlation we found, between cynomolgus and human MDR1, is still relatively good (R^2^ = 0.75), confirming previous findings that overall substrate recognition is conserved across species [15,49,55,56,57,58]. A few studies, however, showed moderate differences between human, rat and mouse [14,55,59,60,61]. Examples include differences in substrate susceptibility between human and mouse for phenytoin and levetiracetam [16,62] or a GSK discovery compound being a rat but not human MDR1 substrate [10].

Our results indicate that the dynamic range is different across cell lines; therefore, resolution efficiency for weaker substrates also varies [63,64]. The largest dynamic range and transporter abundance were found in hMDR1 cells [62,63]. However, there is no apparent correlation between rank order in MDR1 quantity and resolution efficiency in the other three cell lines (Table S1). This was apparent after normalization for transporter abundance, which changed the slope of correlation as well as deviation from the line of identity [54,62], highlighting that although transport rate is linked to MDR1 abundance, the kinetic parameters need to be taken into account in in vitro measurements. A few compounds deviated from the correlation line, suggesting potential species differences. Since the usefulness of an in vitro system depends on its predictive performance and translatability [53], ERs in our cells were compared to rodent K_p,uu,brain_ data. The best sensitivity (85%) was achieved with the hMDR1 cells, likely because it has the highest transporter expression, in agreement with studies comparing the two widely used MDCK-MDR1 cell lines, NIH and Borst [26,42,63,65]. Our hMDR1 cells excel because of (a) the lack of endogenous MDR1 expression, and (b) their high human transporter expression and corresponding ability to recognize weak substrates [29,42,66,67]. Using total ERs derived from MDR1 and BCRP cells further increased predictive power, even compared to other cell lines evaluated for CNS penetration classification [42]. This holds up even when compared to double-transfected cells, likely due to the relatively high but comparable expression level of the two transporters in our cell lines [27,68,69,70]. It follows that a combination screening of MDR1 and BCRP can improve prediction performance, but dual overexpression at high levels in the same cell is technically challenging. In addition to BBB penetration classification, in vitro data can be used for the quantitative prediction of K_p,uu,brain_ [28,29,42,71]. We compared calculations using hMDR1 and total ERs, REF-corrected total ERs, and a mathematically parametrized calculation. The predictive performance was increased accordingly, with the best correlation (R^2^ = 0.83) between in vivo and predicted K_p,uu,brain_ achieved with scaling factors.

Studies on species-related differences in MDR1 inhibition are scarce [15,72,73]; therefore, we aimed to further investigate this phenomenon in our MDR1 cells. The selection of substrates is a key consideration when establishing an in vitro inhibition assay [74,75]. As almost the entire inner surface of MDR1 can interact with the ligand, it is difficult to identify a genuine affinity site. Digoxin is the most common clinical probe for MDR1 DDI studies, and a sensitive in vitro probe with high ER but relatively low affinity towards MDR1 (ICH, M12), [76,77,78,79]. As digoxin is polar, active uptake and partitioning into the membrane might be rate-limiting for its interaction with MDR1 [80]; its IC_50_ therefore represents multiple transport processes [76]. In contrast, quinidine is amphiphilic with moderate permeability and higher affinity [81], and an MDR1 inhibitor as well [82,83]. Quinidine is thought to be transported across the cells solely by MDR1 and passive permeability [84,85]. In our cells, higher hMDR1 expression likely contributed to the larger dynamic range; still, digoxin—but not quinidine—ERs were comparable across cell lines, with quinidine ER values 3–4-fold higher in hMDR1 than in orthologs. This discrepancy was also seen when comparing IC_50_s across orthologs and substrates. IC_50_ values of non- human orthologs with digoxin were generally much lower compared to those of hMDR1, while this was not typical with quinidine. Although hMDR1 featured higher digoxin IC_50_s, digoxin DDI risk was correctly predicted using this cell line when implementing the regulatory agency-recommended static model [35]. Despite several notable differences in the absolute IC_50_s between the two substrates, rank order profiles were generally similar. Testing the inhibition potential of the two substrates against each other resulted in low IC_50_ for cynomolgus and rat, and high IC_50_ values for human and mouse MDR1 when using quinidine as the inhibitor [72]. However, digoxin did not inhibit quinidine transport, confirming its classification as a substrate but not an inhibitor of MDR1 [86,87,88]. All inhibitors interacted with the MDR1, except talinolol and ritonavir. Although talinolol is a known MDR1 substrate [89], in accordance with others [35], our results support the lack of clinical interaction with digoxin [90]. Despite its known effect on digoxin pharmacokinetics [91,92], ritonavir’s low permeability prevents studying its interaction with MDR1 in vectorial transport assays [93]. However, ritonavir DDI potential was shown in another assay [94]. To reveal the IC_50_’s dependence on substrate, transporter abundance or orthologs, patterns in IC_50_s were recognized. The first group of inhibitors, comprising elacridar, ketoconazole and zosuquidar, had comparable IC_50_s in all MDR1 assays with both substrates, independent of protein abundance. This might be explained by physicochemical properties and the nature of MDR1 interaction, since elacridar and zosuquidar can uncouple ATPase activity from transport [95,96,97,98,99], while ketoconazole is thought to inhibit MDR1 by binding to a central modulatory site [100]. In a second group (etoricoxib, loperamide, nitrendipine, saquinavir and CSA), IC_50_s are proportional to transporter abundance, independently of substrate [101]. Thus, transporter abundance-corrected IC_50_s correlate better. As an example, CSA IC_50_s are proportional to transporter expression, but less dependent on the substrate, consistent with previous findings that CSA [102] is a substrate of MDR1, suggesting a competitive inhibitory mechanism.

In the third group (tolvaptan, isradipine, carvedilol and verapamil), dependence on transporter abundance was apparent only with digoxin and not with quinidine, suggesting different inhibitory mechanisms with the two substrates. Differences in IC_50_s across cell lines with varying MDR1 expression were reported for verapamil [101]. Studies support the simultaneous binding of verapamil and digoxin to MDR1 [78,103], suggesting competitive inhibition. In contrast, verapamil likely displays noncompetitive interaction with quinidine [104].

The mechanism of MDR1 inhibition is still poorly understood, involving multiple binding sites and inhibitory mechanisms. Although our study does not provide information on the mechanism of inhibition per se, it gives new insights into substrate and transporter abundance-dependent inhibition of MDR1. We confirmed that the inhibitory potency of many drugs is highly substrate-dependent, resulting in generally lower IC_50_ with quinidine, despite the good overlap in rank order of inhibitors. Rank order was similar between species as well; however, differences of an order of magnitude were observed in IC_50_s. In some cases, IC_50_ difference was resolved by transporter abundance correction, but this correlation is not always proportional and dependent on substrate-inhibitor interaction, suggesting that the correlation between IC_50_ and MDR1 abundance is dependent on the mode of inhibition.

In summary, species differences in DDI potential can be investigated in this system, but transporter abundance and mode of inhibition need to be considered. Our in vitro assays are ready to be integrated into CNS drug discovery screening programs to predict BBB penetration or reveal potential species differences in transporter susceptibility, improving the translatability of in vivo preclinical data.

## Figures and Tables

**Table 1:** Predictive performance metrics (ER cut-off 2 or 3; in vivo K_p,uu,brain_ cut-off 0.3).

	hMDR1	rMDR1	mMDR1	cyMDR1	Total ER	REF-Corrected Total ER
False Negative (n)	5	6	10	12	3	6
False Positive (n)	2	3	2	1	5	3
True Negative (n)	22	21	22	23	19	21
True Positive (n)	29	28	24	22	31	28
PPV	94%	90%	92%	96%	86%	90%
NPV	81%	78%	69%	66%	86%	78%
Sensitivity	85%	82%	71%	65%	91%	82%
Specificity	92%	88%	92%	96%	79%	88%
Accuracy	88%	84%	79%	78%	86%	84%
FN Rate	15%	18%	29%	35%	9%	18%

Positive predictive values (PPV) were calculated by dividing the number of true positives by the total number of true and false positives. Similarly, negative predictive values (NPV) were calculated as the number of true negatives divided by the total number of true and false negatives. Assay sensitivity was calculated by dividing true positives by the total number of false negatives and true positives, whereas specificity was determined as true negatives divided by the total number of false positives and true negatives. Assay accuracy was calculated by dividing true positives and true negatives by the total number of studies.

**Table 2:** Estimated α and β parameters with confidence intervals using Equation (9).

Parameter	Estimate	Standard Error	Lower 95% CI	Upper 95% CI
*α*	0.52	0.103	0.315	0.731
*β*	0.29	0.080	0.126	0.450

## Data Availability

The data presented in this study are available on request from the corresponding author. The data are not publicly available due to company participation.

## Supplementary Materials

The following supporting information can be downloaded at: https://www.mdpi.com/article/10.3390/pharmaceutics16060736/s1, Figure S1: Residual plots of the fitted model (Equation (9)) for 55 tested compounds according to Table S1; Figure S2: Residual plots of the fitted model (Equation (9)) with exclusion of compounds with K_p,uu,brain_ >1.2 according to Table S1; Table S1: ERs across the six cell lines for 83 compounds (1 µM, 120 min); Table S2: Summary of transporter protein abundance in isolated brain capillaries (mouse, rat, cynomolgus monkey and human) in Abcb1KO-MDCKII-MDR1 and in MDCKII-BCRP cell lines; Table S3: IC_50_ values when investigating inhibition of hMDR1, rMDR1, mMDR1 and cyMDR1 transport of either digoxin or quinidine by a range of marketed compounds
