# Supplementary material for: Applicability of MDR1 Overexpressing Abcb1KO-MDCKII Cell Lines for Investigating In Vitro Species Differences and Brain Penetration Prediction
Source: Pharmaceutics. 2024 May 29;16(6):736. doi: 10.3390/pharmaceutics16060736 (PMC11207571; doi:10.3390/pharmaceutics16060736)
Supplement: Supplementary file 1 [file pharmaceutics-16-00736-s001.zip › pharmaceutics-3015208-supplementary.pdf]

SUPPLEMENTARY INFORMATION

Applicability of MDR1 overexpressing Abcb1KO-MDCKII cell lines for investigating in vitro species differences and brain penetration prediction

Emőke Sóskuti, Nóra Szilvásy, Csilla Temesszentandrási-Ambrus, Zoltán Urbán, Olivér Csíkvári, Zoltán Szabó, Gábor Kecskeméti, Éva Pusztai, Zsuzsanna Gáborik

SUPPLEMENTARY TABLES

Table S1. Efflux Ratios across the six cell lines for 83 compounds (1 µM, 120 min)

Compounds	Efflux Ratios in Abcb1KO-MDCKII					ER in MDCKII
	Mock	hMDR1	rMDR1	mMDR1	cyMDR1	BCRP
Acrivastine	0.58 ± 0.05	5.49 ± 0.23	1.94 ± 0.18	1.55 ± 0.14	1.17 ± 0.14	7.37 ± 2.01
Amiodarone*	0.36 ± 0.04	0.71 ± 0.09	0.41 ± 0.05	0.41 ± 0.07	2.51 ± 0.26	3.13 ± 1.74
Amisulpride	1.19 ± 0.07	18.68 ± 1.92	10.52 ± 1.16	11.82 ± 0.85	6.22 ± 0.32	28.92 ± 3.47
Amitriptylline*	1.05 ± 0.08	1.1 ± 0.11	0.94 ± 0.14	0.96 ± 0.12	0.67 ± 0.08	0.8 ± 0.07
Amprenavir	0.63 ± 0.03	69.53 ± 4.08	36.06 ± 2.46	31.95 ± 0.96	24.88 ± 1.15	4.16 ± 0.37
Antipyrine	0.65 ± 0.02	0.75 ± 0.05	0.65 ± 0.03	0.66 ± 0.04	0.67 ± 0.02	0.84 ± 0.02
Astemizole*	1.43 ± 0.24	12.25 ± 1.29	3.27 ± 0.73	4.52 ± 0.85	10.62 ± 1.49	13.55 ± 2.46
Atenolol	1.4 ± 0.55	1.8 ± 0.2	1.22 ± 0.16	1.37 ± 0.19	1.17 ± 0.08	1.34 ± 0.12
Atrazine	0.52 ± 0.04	0.52 ± 0.04	0.46 ± 0.04	0.59 ± 0.1	0.54 ± 0.03	0.63 ± 0.02
Bepotastine	1.62 ± 0.17	9.51 ± 0.45	8.86 ± 1.16	10.03 ± 1.31	7.91 ± 1.59	2.19 ± 0.3
Bromocriptine*	0.68 ± 0.07	4.85 ± 0.39	1.75 ± 0.09	1.39 ± 0.1	1.08 ± 0.67	0.52 ± 0.05
Caffeine	0.76 ± 0.03	0.81 ± 0.05	0.8 ± 0.05	0.77 ± 0.03	0.87 ± 0.05	1.11 ± 0.02
Carbamazepine	0.61 ± 0.06	0.62 ± 0.06	0.86 ± 0.08	0.79 ± 0.07	0.75 ± 0.07	0.86 ± 0.12
Carvedilol	0.62 ± 0.04	14.47 ± 0.62	2.13 ± 0.08	1.94 ± 0.21	1.12 ± 0.06	2.38 ± 0.23
Cetirizine	0.75 ± 0.03	17.69 ± 0.63	10.55 ± 0.62	6.96 ± 0.39	4.72 ± 0.21	2.43 ± 0.14
Chlorpromazine*	1.39 ± 0.14	0.61 ± 0.1	1.4 ± 0.18	0.59 ± 0.06	1.06 ± 0.2	0.43 ± 0.11
Cimetidine	1.42 ± 0.19	6.98 ± 0.72	9.86 ± 0.98	2.83 ± 0.37	2.75 ± 0.32	72.55 ± 11.46
Clarithromycin	0.53 ± 0.02	96.25 ± 3.83	25.05 ± 5.14	27.52 ± 1.62	17.16 ± 3.25	11.12 ± 1.46
Clonidine	0.93 ± 0.03	0.78 ± 0.02	0.98 ± 0.05	0.99 ± 0.06	0.8 ± 0.05	0.89 ± 0.04
Daunorubicin	1.04 ± 0.08	21.7 ± 5.36	16.86 ± 1.99	27.74 ± 2.63	83.08 ± 23.16	62.53 ± 14.06
Desloratadine	0.93 ± 0.1	49.56 ± 2.29	39.93 ± 3.12	25.7 ± 1.26	19.87 ± 1.39	2.04 ± 0.16
Dexamethasone	0.45 ± 0.06	8.41 ± 1.05	8.36 ± 0.67	5.38 ± 0.5	3.41 ± 0.49	3.39 ± 0.44
Dextromethorphan	0.98 ± 0.1	1.03 ± 0.08	0.94 ± 0.09	0.78 ± 0.05	0.75 ± 0.06	0.66 ± 0.08
Digoxin	1.08 ± 0.07	17.99 ± 1.56	21.94 ± 1.43	19.2 ± 1.59	16.32 ± 1.83	8.58 ± 0.68
Diltiazem	0.68 ± 0.04	3.2 ± 0.3	1.64 ± 0.07	1.02 ± 0.13	0.83 ± 0.08	0.19 ± 0.04
Disopyramide	0.71 ± 0.12	34.72 ± 2.68	21.09 ± 3.7	11.24 ± 0.8	17.17 ± 3.18	4.2 ± 1.09
Domperidone	0.8 ± 0.16	91.04 ± 11.96	75.23 ± 8.78	54.41 ± 2.74	24.67 ± 2.62	40.03 ± 6.96
Donepezil	1.22 ± 0.05	1.79 ± 0.13	1.44 ± 0.11	1.29 ± 0.08	1.17 ± 0.05	0.75 ± 0.06
Doxepin	0.68 ± 0.09	0.79 ± 0.13	1.09 ± 0.16	0.91 ± 0.11	0.71 ± 0.06	0.54 ± 0.07
Doxylamine	0.83 ± 0.03	1.68 ± 0.04	1.39 ± 0.03	1.25 ± 0.06	0.96 ± 0.06	0.97 ± 0.09
Entacapone	0.32 ± 0.02	0.28 ± 0.02	0.39 ± 0.03	0.34 ± 0.05	0.46 ± 0.07	49.44 ± 3.92
Etoposide	0.67 ± 0.08	11.23 ± 0.8	5.98 ± 0.85	6.87 ± 1.42	4.41 ± 0.91	14 ± 2.31
Etoricoxib	0.9 ± 0.09	1.97 ± 0.21	2.01 ± 0.21	1.72 ± 0.23	1.24 ± 0.21	2.35 ± 0.25
Felodipine*	0.29 ± 0.02	0.32 ± 0.02	0.26 ± 0.02	0.32 ± 0.02	0.34 ± 0.02	0.84 ± 0.04
Fluoxetine *	0.4 ± 0.06	0.62 ± 0.03	0.26 ± 0.02	0.42 ± 0.06	0.36 ± 0.04	0.54 ± 0.05
Guanabenz	0.93 ± 0.15	0.82 ± 0.1	0.85 ± 0.13	1.08 ± 0.09	0.84 ± 0.1	3.35 ± 1.17
Gefitinib	0.93 ± 0.11	30.54 ± 1.66	9.86 ± 0.85	8.98 ± 0.82	4.65 ± 0.62	45.09 ± 7.77
Guanfacine	0.63 ± 0.06	2.04 ± 0.21	0.97 ± 0.12	1.62 ± 0.16	0.65 ± 0.11	0.32 ± 0.08
Haloperidol	0.74 ± 0.1	0.89 ± 0.22	0.85 ± 0.15	0.72 ± 0.11	0.8 ± 0.22	0.72 ± 0.1
Imatinib	0.99 ± 0.11	60.22 ± 4.74	29.07 ± 1.26	23.06 ± 1.52	10.48 ± 0.6	49.21 ± 0.99
Imipramine	0.62 ± 0.04	1.08 ± 0.05	0.83 ± 0.08	0.9 ± 0.05	0.82 ± 0.09	0.6 ± 0.06
Indinavir	0.93 ± 0.31	33.23 ± 1.69	10.87 ± 1.21	11.16 ± 2.53	34.77 ± 10.41	29.7 ± 3.69
Indomethacin	0.37 ± 0.07	0.42 ± 0.05	0.4 ± 0.01	0.39 ± 0.02	0.4 ± 0.03	10.16 ± 0.71
Ketoconazole	1.24 ± 0.06	5.45 ± 0.34	3.47 ± 0.17	3.13 ± 0.18	2.69 ± 0.15	0.79 ± 0.12
Lamotrigine	0.79 ± 0.05	0.8 ± 0.05	1.07 ± 0.08	0.81 ± 0.04	0.82 ± 0.05	1.38 ± 0.17
Levofloxacin	0.59 ± 0.02	2.32 ± 0.13	2.07 ± 0.08	1.57 ± 0.13	0.85 ± 0.03	8.16 ± 0.55
Lidocaine	0.84 ± 0.02	0.91 ± 0.08	0.93 ± 0.03	0.87 ± 0.06	0.95 ± 0.04	0.8 ± 0.04
Loperamide	0.49 ± 0.03	48.73 ± 3.51	10.46 ± 2.34	16.01 ± 2.75	6.38 ± 0.55	2.00 ± 0.23
Mannitol	0.96 ± 0.12	0.87 ± 0.14	0.89 ± 0.12	1.04 ± 0.16	0.76 ± 0.1	0.73 ± 0.2
Metoclopramide	0.85 ± 0.07	5.55 ± 0.43	3.02 ± 0.28	2.55 ± 0.21	1.44 ± 0.14	2.42 ± 0.33
Metoprolol	0.74 ± 0.05	1.53 ± 0.15	0.97 ± 0.11	0.92 ± 0.08	0.74 ± 0.05	0.71 ± 0.11
Mexiletine	0.36 ± 0.05	0.44 ± 0.03	0.42 ± 0.08	0.64 ± 0.16	0.51 ± 0.15	0.71 ± 0.05
Mibefradil*	1.06 ± 0.13	29.7 ± 3.7	6.48 ± 0.87	13.92 ± 2.03	5.69 ± 1.4	0.25 ± 0.07
Midazolam	0.43 ± 0.04	0.53 ± 0.03	0.28 ± 0.06	0.4 ± 0.06	0.43 ± 0.05	0.57 ± 0.06
Mitoxantrone	1.15 ± 0.14	5.66 ± 0.54	17.62 ± 1.56	28.56 ± 4.05	40.26 ± 2.47	55.57 ± 10.79
Naloxone	0.33 ± 0.06	0.36 ± 0.04	0.61 ± 0.1	0.37 ± 0.03	0.48 ± 0.11	0.86 ± 0.05
Naltrexone	0.88 ± 0.05	1.13 ± 0.07	1.55 ± 0.13	1.35 ± 0.16	1.17 ± 0.17	0.86 ± 0.05
Nelfinavir*	0.79 ± 0.09	101.59 ± 6.11	50.45 ± 3.63	31.47 ± 3.06	31.56 ± 2.29	1.45 ± 0.12
Ondansetron	0.7 ± 0.02	5.24 ± 0.68	7.62 ± 0.39	5.33 ± 0.54	3.77 ± 0.64	4.28 ± 0.71
Paclitaxel	2.52 ± 0.51	40.21 ± 12.58	82.89 ± 41.14	57.68 ± 17.01	33.36 ± 5.69	63.77 ± 28.48
Paliperidone	0.79 ± 0.1	19.77 ± 2.06	6.06 ± 1.45	8.26 ± 0.88	2.47 ± 0.41	5.82 ± 1.79

**Pheniramine**	0.78 ± 0.04	1.45 ± 0.05	1.09 ± 0.03	1.1 ± 0.1	0.99 ± 0.02	1.02 ± 0.03
**Prazosin**	0.72 ± 0.03	9.35 ± 0.45	6.5 ± 0.22	5.14 ± 0.39	2.42 ± 0.15	14.79 ± 1.68
**Prednisone**	0.82 ± 0.11	17.26 ± 2.38	9.6 ± 1.82	6.6 ± 0.57	4.41 ± 0.52	1.33 ± 0.1
**Propafenone**	0.62 ± 0.07	6.19 ± 0.73	1.59 ± 0.2	1.64 ± 0.14	0.92 ± 0.07	0.88 ± 0.08
**Propranolol**	0.54 ± 0.06	0.88 ± 0.08	0.73 ± 0.08	0.92 ± 0.1	0.66 ± 0.06	0.71 ± 0.15
**Quinidine**	1.21 ± 0.06	48.23 ± 4.07	20.05 ± 1.47	20.65 ± 1.87	8.98 ± 0.77	3.38 ± 0.37
**Ranitidine**	0.91 ± 0.06	2.89 ± 0.15	2.63 ± 0.19	2.09 ± 0.3	2.14 ± 0.25	10.35 ± 0.96
**Risperidone**	0.52 ± 0.04	3.4 ± 0.52	2.38 ± 1	2.2 ± 0.31	1.23 ± 0.36	0.94 ± 0.13
**Ritonavir**	0.82 ± 0.06	66.19 ± 16.49	90.79 ± 11.91	75.44 ± 8.25	54.7 ± 8.1	23.93 ± 2.1
**Saquinavir**	0.77 ± 0.06	31.24 ± 2.66	50.64 ± 4.64	47.73 ± 3.46	75.42 ± 15.39	68.48 ± 13.49
**Sertraline***	3.57 ± 0.3	3.1 ± 0.34	2.74 ± 0.28	2.93 ± 0.43	5.65 ± 0.81	3.7 ± 0.67
**Sumatriptan**	0.93 ± 0.04	1.88 ± 0.12	2.02 ± 0.12	1.76 ± 0.08	1.53 ± 0.1	1.74 ± 0.24
**Tacrine**	0.81 ± 0.02	0.93 ± 0.04	0.88 ± 0.02	0.99 ± 0.03	0.83 ± 0.02	1.19 ± 0.07
**Tamoxifen***	2.25 ± 1.24	1.65 ± 0.24	1.61 ± 0.18	2.26 ± 0.76	1.81 ± 0.51	0.81 ± 0.14
**Telmisartan**	0.38 ± 0.06	9.86 ± 2.27	2.65 ± 0.75	2.2 ± 0.27	3.18 ± 0.63	12.24 ± 0.95
**Terfenadine***	0.88 ± 0.06	10.72 ± 0.78	1.29 ± 0.14	2.64 ± 0.33	0.92 ± 0.14	2.96 ± 0.31
**Trazodone**	2.66 ± 0.59	1.71 ± 0.34	1.4 ± 0.16	0.98 ± 0.24	1.5 ± 0.1	0.86 ± 0.11
**Trimethoprim**	0.84 ± 0.06	7.29 ± 0.69	13.24 ± 0.68	9.04 ± 0.49	1.65 ± 0.1	3.08 ± 0.17
**Verapamil**	1.12 ± 0.04	6.81 ± 0.28	2.93 ± 0.13	2.46 ± 0.22	1.73 ± 0.09	0.89 ± 0.05
**Vinblastine**	1.32 ± 0.28	51.58 ± 5.3	61.19 ± 5.31	50.5 ± 6.89	61.4 ± 7.47	22.9 ± 4.47
**Warfarin**	0.54 ± 0.02	0.56 ± 0.02	0.6 ± 0.01	0.58 ± 0.02	0.58 ± 0.05	4.03 ± 0.29
**Zolmitriptan**	0.97 ± 0.09	4.3 ± 0.26	2.69 ± 0.16	1.99 ± 0.16	2.24 ± 0.22	3.94 ± 0.25
**Famotidine#**	1.05 ± 0.04	1.08 ± 0.04	1.08 ± 0.09	0.97 ± 0.06	0.94 ± 0.15	0.59 ± 0.23

*Excluded from data analysis due to low recovery (≤ 60%)

Famotidine was used as permeability control, it was not included in the correlation analyses.

Red colour shows impared CNS penetration ($K_{p,uu,brain} \leq 0.3$), green colour shows CNS penetrant compounds ($K_{p,uu,brain} > 0.3$). The colour of the compounds refer to the in vivo rodent $K_{p,uu,brain}$ data. The colour of MDR1 ERs refer to the predicted REF-corrected $K_{p,uu,brain}$ for the individual cell lines (according to equation 6). The colours of BCRP column refer to predicted $K_{p,uu,brain}$ from total ERs with α and β scaling factors (according to equation 9).

Table S2. Summary of transporter protein abundance in isolated brain capillaries (mouse, rat, cynomolgus monkey and human) in Abcb1KO-MDCKII-MDR1 and in MDCKII-BCRP cell lines

	**Transporter protein abundance (pmol/mg)**				
	**MDR1**				**BCRP**
	**human**	**rat**	**mouse**	**cynomolgus monkey**	**human**
**Isolated brain capillaries**x	5.42[4,19]	19.13[4,6,7,18]	15.05[4,17]	5.36[4,5]	8[4]
**MDCKII cell lines**	25.14	7.09	12.08	13.64	8.9

xdata were taken from previously published studies.

Table S3. IC_{50} values when investigating inhibition of hMDR1, rMDR1, mMDR1 and cyMDR1 transport of either digoxin or quinidine by a range of marketed compounds

		**Inhibition of digoxin transport**				**Inhibition of quinidine transport**			
		**IC_{50}±SD (µM)**				**IC_{50}±SD (µM)**			
**Nr**	**INHIBITOR**	**hMDR1**	**rMDR1**	**mMDR1**	**cyMDR1**	**hMDR1**	**rMDR1**	**mMDR1**	**cyMDR1**
**1**	**Carvedilol**	19.13±7.45	4.22±1.00	2.00±0.55	1.55±0.59	0.41±0.24	1.01±0.13	1.64±0.90	1.02±0.79
**2**	**Cyclosporin A**	6.44±0.81	0.33±0.11	0.13±0.04	0.17±0.06	1.19±0.00	0.12±0.07	0.22±0.13	0.23±0.08
**3**	**Quinidine/ Digoxin**	37.47±16.78	2.93±1.43	15.00±4.06	0.80±0.24	NI	NI	NI	NI
**4**	**Elacridar**	0.35±0.15	0.32±0.15	0.10±0.07	0.09±0.03	0.13±0.07	0.06±0.00	0.20±0.04	0.11±0.08
**5**	**Etoricoxib**	> 100 µM	54.64±3.16	57.84±29.79	15.49±11.66	> 100 µM	18.81±1.22	22.17±5.43	25.93±10.49
**6**	**Felodipine**	> 100 µM	> 100 µM	28.64±7.95	12.43±5.74	> 100 µM	40.6±7.71	> 100 µM	50.65±19.66

**7**	**Isradipine**	> 50 µM	15.17±2.84	5.28±2.94	10.60±4.73	2.07±1.28	13.61±9.37	19.34±3.75	19.34±5.76
**8**	**Itraconazole**	9.77±1.26	4.41±1.05	1.39±0.50	0.36±0.19	0.14±0.05	0.15±0.07	0.48±0.11	0.32±0.12
**9**	**Ketoconazole**	4.65±1.21	3.74±1.55	2.65±1.57	2.52±1.16	0.72±0.00	1.12±0.00	0.32±0.03	0.81±0.28
**10**	**Loperamide**	38.25±4.59	11.05±4.28	6.50±1.10	6.30±2.27	4.75±0.32	1.01±0.49	0.96±0.33	1.36±0.34
**11**	**Mibefradil**	12.97±5.13	7.42±2.76	7.15±4.58	4.84±1.82	0.91±0.38	1.11±0.21	1.55±062	3.61±2.04
**12**	**Nitrendipine**	> 50 µM	4.02±1.64	> 50 µM	3.15±1.70	> 50 µM	6.34±2.68	15.73±5.68	17.54±4.96
**13**	**Propafenone**	44.76±20.41	6.23±1.15	21.40±1.85	0.30±0.17	0.78±0.59	1.71±0.17	1.99±1.07	1.70±0.80
**15**	**Saquinavir**	> 50 µM	25.01±11.40	29.23±9.84	12.29±5.44	> 50 µM	12.25±5.02	11.22±5.12	20.67±7.67
**17**	**Telmisartan**	15.22±5.66	11.63±3.91	2.60±1.59	4.06±1.69	1.81±0.87	2.72±1.26	2.28±0.97	1.55±0.53
**18**	**Tolvaptan**	> 30 µM	8.79±2.81	10.92±1.46	6.68±1.63	1.28±0.44	4.14±1.47	3.92±0.87	5.11±1.65
**19**	**Verapamil**	29.83±7.06	2.00±0.71	1.86±0.71	0.86±0.12	0.91±0.47	8.47±2.26	1.19±0.54	1.42±0.31
**20**	**Zosuquidar**	0.10±0.02	0.60±0.29	0.37±0.13	0.05±0.02	0.05±0.04	0.57±0.21	0.49±0.26	0.12±0.09

Ritonavir (14) and **talinolol** (16) showed no inhibition on MDR1-mediated transport in these assays.

SUPPLEMENTARY FIGURES

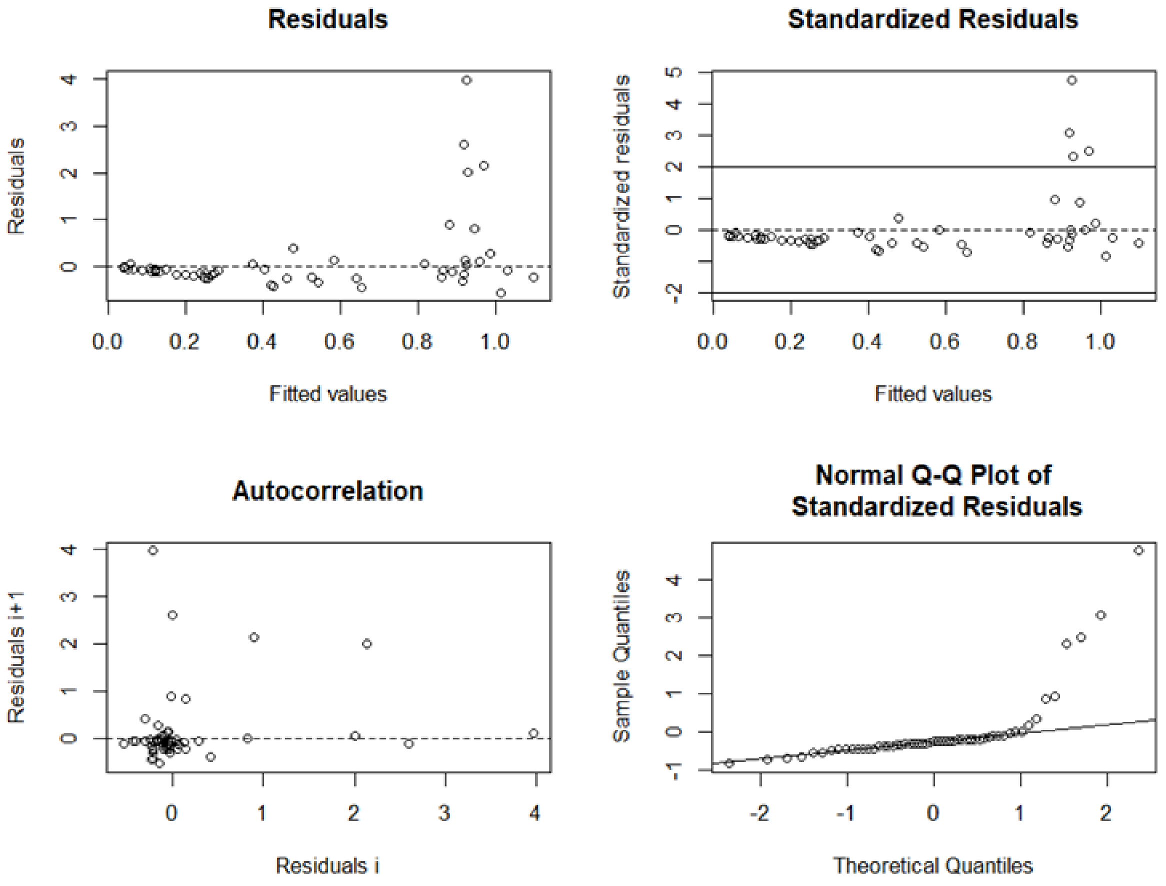

Figure S1: Residual plots of the fitted model (Eq. 9) for 55 tested compounds according to Table S1.

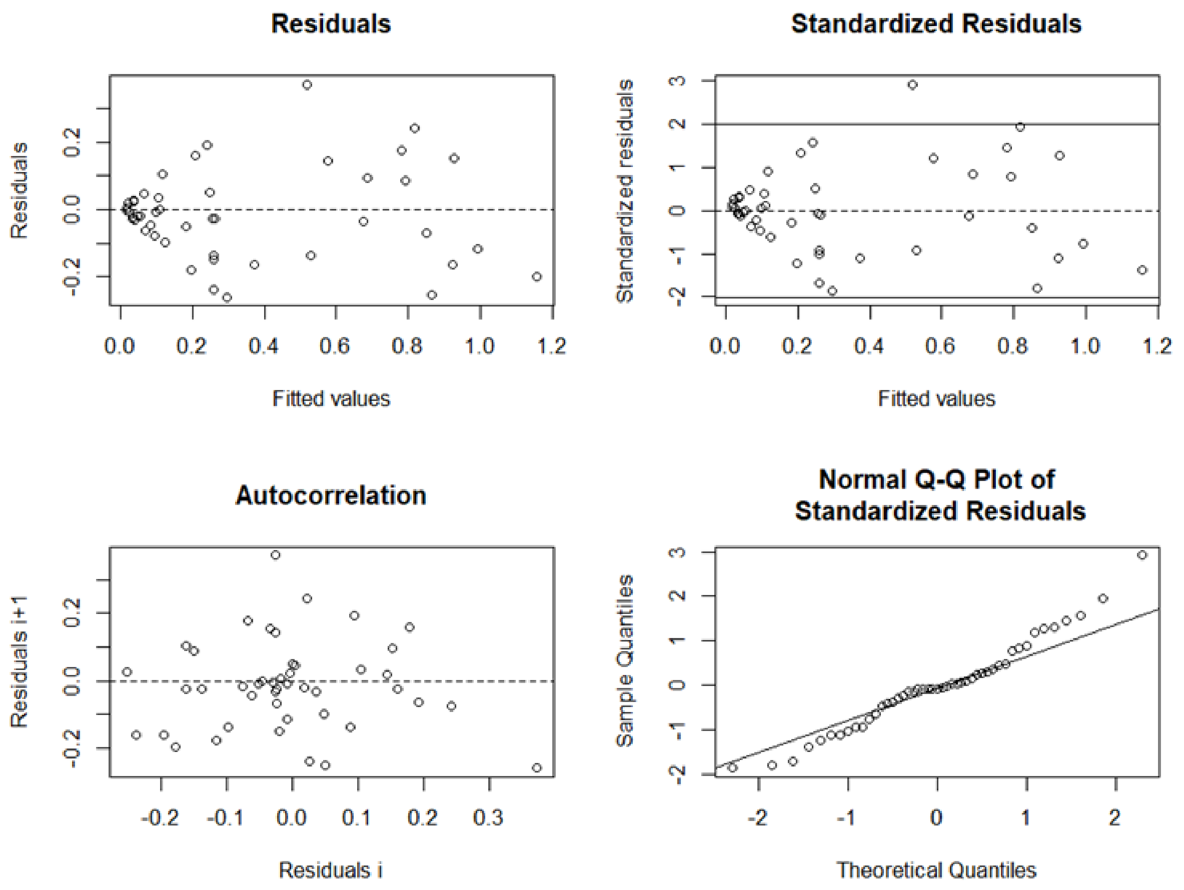

Figure S2: Residual plots of the fitted model (Eq. 9) with exclusion of compounds with $K_{p,uu,\, brain}$ >1.2 according to Table S1.
